# Tricuspid regurgitation after heart transplantation: where innovation and intervention are in hibernation

**DOI:** 10.1007/s10741-025-10494-2

**Published:** 2025-02-13

**Authors:** Emyal Alyaydin, Alexander Gotschy, Danaë Parianos, Matthias P. Nägele, Igor Tudorache, Andreas J. Flammer, Julia Stehli

**Affiliations:** 1 Department of Cardiology, University Hospital Zurich, Zurich, Switzerland; 2 Institute of Diagnostic and Interventional Radiology, University Hospital Zurich, Zurich, Switzerland; 3 Institute for Biomedical Engineering, University and ETH Zurich, Zurich, Switzerland; 4 Clinic for Cardiac Surgery, University Heart Center Zurich, Zurich, Switzerland; 5 Department of Cardiology, St. Johannes Hospital, Dortmund, Germany

**Keywords:** Tricuspid regurgitation, Heart transplantation, Heart valve prosthesis implantation, Transcatheter edge-to-edge repair

## Abstract

Tricuspid regurgitation (TR) is a common valvular dysfunction following heart transplantation (HT), with reported prevalence rates ranging from 19 to 84%, primarily depending on the duration of follow-up. Its etiology is multifactorial and includes surgical technique, ischemic time, primary graft dysfunction, pulmonary hypertension, pacemaker leads, and endomyocardial biopsies. Severe TR can significantly impair graft function, exercise capacity, and patient survival. This mini-review explores current management strategies, including surgical techniques such as annuloplasty and tricuspid valve replacement. HT patients are often high-risk surgical candidates due to factors such as immunosuppressive therapy, prior surgeries, and various comorbidities. Therefore, we primarily focus on the evidence regarding emerging interventional methods, such as transcatheter edge-to-edge repair. Although these interventions show promising early results, they remain relatively novel in HT recipients, and the current evidence is based on case reports and small studies. Further research is essential to evaluate the long-term efficacy and safety of these management strategies to enhance outcomes for HT recipients with TR.

**Graphical Abstract:**

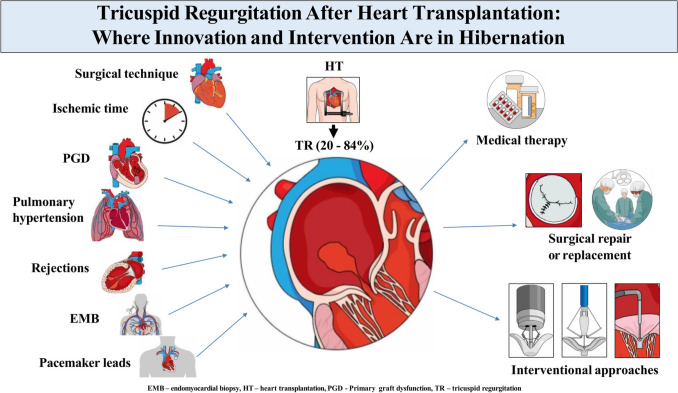

## Introduction

Tricuspid regurgitation (TR) is the most common valvular dysfunction after heart transplantation (HT). The reported prevalence of TR in HT recipients varies widely, with a range between 19 and 84% [1, 2]. This variability is influenced by the duration of follow-up and the severity of TR, with milder forms being more common, although severe TR is also observed in a significant subset of patients [3, 4]. The etiology of TR is multifactorial, encompassing factors such as surgical technique, donor heart function, ischemic time, and particularly valvular damage during endomyocardial biopsy (EMB) [5–7]. Severe TR can influence graft function, exercise tolerance, and patient survival in the short- and long-term follow-up [8–12].

This review aims to highlight the current evidence on TR following HT, considering recent advancements in interventional approaches. A thorough understanding of these advances is crucial for assessing the clinical consequences of TR on patient outcomes.

## Etiologic considerations

### Surgical technique

The choice of surgical technique during HT—biatrial (Shumway technique), bicaval, or total—significantly affects the incidence and severity of TR. Studies have demonstrated that biatrial anastomosis, which involves connecting the atria of the donor and the recipient, is associated with a significantly higher prevalence of post-transplant TR due to its impact on right atrial geometry and function [2, 3]. In contrast, bicaval anastomosis involves performing a standard left atrial anastomosis and a separate bicaval anastomosis, where both the superior and inferior vena cavae of the donor heart are individually connected to the recipient’s corresponding veins, thereby avoiding the described right atrial challenges [9, 13]. The total anastomosis technique, although less commonly employed, is a more advanced approach. It includes the full excision of the recipient atria, along with complete atrioventricular transplantation and individual bicaval and pulmonary venous anastomoses. The main drawbacks of this technique are the longer time required for six anastomoses and the increased risk of complications, such as bleeding from posterior pulmonary vein sutures and pulmonary venous stenosis [13]. Research consistently shows that the bicaval and total technique are associated with a lower incidence of severe TR and improved long-term outcomes compared to the biatrial approach [5, 9].

### Ischemic time of the donor heart

Ischemic time of the donor heart critically influences TR after HT. Prolonged ischemia can aggravate myocardial injury and impair valve function, leading to a higher incidence of TR. Extended ischemic periods correlate with increased myocardial damage and poorer valvular outcomes [12, 14].

### Primary graft dysfunction (PGD)

PGD is a significant factor contributing to TR following HT. This is a condition where the donor heart cannot meet the recipient’s circulatory demands immediately after the transplant due to either single or biventricular dysfunction. It presents as hypotension and low cardiac output despite adequate filling pressures.

PGD can lead to impaired right ventricular function and elevated pulmonary pressures, both of which exacerbate TR. Patients with PGD may develop TR, as the compromised graft function increases afterload on the right ventricle and can cause or worsen valve incompetence [1, 15]. Effective management of PGD is crucial to mitigate the risk and severity of TR and improve long-term transplant outcomes.

### Rejections

Rejection episodes following HT can significantly contribute to the development of TR [15]. Studies highlight that TR associated with rejection correlates with ventricular dysfunction and carries a higher mortality risk compared to other etiologies. The timing of rejection-induced TR is typically triphasic, occurring early post-transplant, mid-term, and long-term, emphasizing its dynamic nature and impact on patient outcomes [16].

#### EMB

EMB is also a notable contributor to TR. This diagnostic procedure, essential for assessing graft rejection, can unintentionally induce or exacerbate TR due to localized trauma to the tendons or leaflets or fibrosis at the tricuspid valve annulus. During tricuspid valve replacement surgeries, ruptured chordae tendineae were identified in nearly all cases, supporting the notion that severe TR can be induced by EMB. The chordae tendineae, particularly those extending from the septal wall, are especially susceptible to trauma from the bioptome. The association between EMB and TR highlights the need for precise procedural management and vigilant follow-up, as persistent TR can negatively impact long-term transplant outcomes [8, 10]. Therefore, while EMB is crucial for graft surveillance, careful procedural techniques and expertise are of major importance to minimize adverse effects. Center volume and operator experience play an important role in the risk of pericprocedural tricuspid injury. In a recent study from a high-volume center, the risk of tricuspid injury was low (0.1% of 1368 biopsies) [17]. Additionally, emerging noninvasive imaging techniques, such as cardiovascular magnetic resonance (CMR), offer a promising alternative for surveillance of acute rejection. CMR-based myocardial tissue characterization, particularly T2 mapping, has shown high diagnostic accuracy and may reduce the need for EMB, decreasing associated risks [18, 19].

### Pulmonary pressures

Immediately after weaning from cardiopulmonary bypass, the transplanted heart is exposed to the recipient’s pulmonary pressures [1]. Increased pulmonary artery pressures impose higher afterload on the right ventricle, which can exacerbate TR by promoting right ventricular dilation and dysfunction. Higher pulmonary pressures are associated with more severe TR. Studies show that pulmonary hypertension can contribute to TR and worsen the condition in those already affected [2, 3, 5]. Managing pulmonary hypertension effectively is therefore crucial in mitigating the development and progression of TR post-transplant.

### Pacemaker leads

Pacemaker implantation, which is required in up to one-fourth of transplant recipients in long-term follow-up, can contribute to TR through lead placement and subsequent impingement of the leaflets [1, 20].

## Diagnosis considerations

TR in OHT recipients presents unique diagnostic challenges, as the criteria for grading and diagnosis traditionally rely on guidelines established for the general population. The altered atrial geometry and changes in ventricular function, often related to rejection episodes or graft adaptation, may limit the applicability of standard grading criteria in this population. Furthermore, while the 2021 ESC/EACTS Guidelines offer comprehensive criteria for TR assessment, these are primarily based on the general population, where the etiology of TR differs significantly [21]. In OHT recipients, TR is often evaluated using color Doppler imaging, but grading schemes vary across studies, with inconsistent use of three- or four-grade systems [22, 23]. These inconsistencies highlight the need for standardized diagnostic criteria tailored specifically to HT patients.

To date, the assessment of TR has primarily been performed using echocardiography. However, CMR is emerging as a valuable tool for determining suitable interventional strategies. It effectively overcomes the limitations of echocardiography, offering detailed morphological evaluations of the tricuspid valve and accurate quantification of TR severity using advanced methods such as indirect volumetric analysis and 4D flow imaging. Furthermore, CMR enables the assessment of right ventricular and atrial remodeling, the quantification of volumes and function without geometrical assumptions, and the provision of tissue characterization. These insights are vital for tailoring therapeutic interventions and optimizing outcomes in patients with TR [24].

### Early and late TR

Early and late TR in HT recipients present distinct patterns and clinical outcomes. In the study by Bishawi et al., significant TR immediately after transplantation was common, affecting 21% of recipients. Early TR was associated with high-grade allograft rejection, elevated pulmonary pressures, and increased pulmonary vascular resistance. In contrast, risk factors for late TR included the use of standard transplant technique, rejections, and the total number of EMBs. Although the severity of TR improved over time in most cases, it was associated with early postoperative morbidity, including longer hospital stays and higher mortality rates at 30 days and 1 year [10]. Early TR was associated with high-grade allograft rejection, elevated pulmonary pressures, and increased pulmonary vascular resistance. In contrast, risk factors for late TR included the use of standard transplant technique, rejections, and the total number of EMBs [10, 25].

### Primary and secondary TR

Primary and secondary TR also differ in their pathophysiology and causes. Functional TR is typically a result of geometric distortion of the atrioventricular annular ring, dilation, and malcoaptation of the valve leaflets. Factors such as biatrial anastomoses, allograft rejection leading to right ventricular dysfunction, or a size mismatch contribute to its development. Studies have shown that atrial size mismatch and dimensions of the recipient atrium are strongly correlated with the occurrence of functional TR. On the other hand, anatomical TR is directly caused by structural damage to the valve apparatus, such as torn leaflets or ruptured chordae tendineae, often following EMB performed to detect allograft rejection. The risk of anatomical TR is associated with the frequency and technique of EMB, with severe cases often resulting in flail valve components and right heart failure [2]. Current guidelines, including the 2021 ESC/EACTS recommendations, do not provide specific guidance for HT patients with primary or secondary TR. For primary TR with diseased valve leaflets, surgical repair or replacement remains the gold standard. In contrast, transcatheter tricuspid valve interventions are primarily considered for high-risk patients with severely symptomatic secondary TR (functional TR) who are not candidates for surgery [21].

### Follow-up

Regarding the frequency of follow-up, the International Society for Heart and Lung Transplantation guidelines for the care of HT recipients recommend that TR identified intraoperatively and estimated to be moderate or severe (> 2 +) should be re-evaluated using transthoracic echocardiography or transesophageal echocardiography within 24 h post-HT. Close monitoring during the initial postoperative days is advised, with the frequency of subsequent evaluations determined by clinical and hemodynamic factors. For routine echocardiographic surveillance in HT recipients, the guidelines recommend evaluations at 2 weeks, 4 weeks, and then monthly during the first 6 months post-HT. From the sixth to twelfth month after HT, assessments are advised every 2 months. After the first year, echocardiography is suggested every 6 months during the second year and annually thereafter, unless clinical circumstances warrant more frequent monitoring. These recommendations are meant to serve as a general guide, with the frequency of follow-up tailored to the patient’s clinical course, particularly in the presence of complications such as graft dysfunction or significant TR [26]. This individualized approach ensures that the surveillance plan is both patient-centered and adaptable to evolving clinical circumstances and technological advancements.

## Clinical implications

The clinical implications of TR after HT are considerable. Severe TR is associated with a marked decline in exercise capacity and functional status [3, 5]. This decline might impact the quality of life, contributing to increased symptoms of heart failure and diminished overall well-being. Patients with severe TR frequently experience peripheral edema, shortness of breath, fatigue, and other heart failure symptoms and have higher rates of hospitalizations. Furthermore, severe TR is linked to poorer long-term outcomes, underscoring the importance of early and effective management to improve patient prognosis and quality of life [10].

## Management

### Medical therapy

The mainstay of therapy for symptomatic severe TR after HT is medical management. This approach focuses on optimizing patient medication, managing volume overload with diuretics, and addressing any contributing factors, such as arrhythmias or pulmonary hypertension.

Interventional or surgical treatment is generally reserved for a minority of patients in whom TR results in refractory symptoms despite optimal medical therapy. However, patient selection for these interventions remains critical, as the clinical benefit must outweigh the procedural risks in this unique patient population.

### Surgical techniques

#### Tricuspid annuloplasty (TA)

The management of TR after HT involves several surgical options aimed at reducing its severity. Techniques such as Carpentier’s ring annuloplasty, the De Vega technique, and valve replacement are commonly utilized [4, 27, 28].

The Carpentier technique involves the systematic repair of tricuspid valve incompetence by assessment of the valve for any organic lesions, such as leaflet thickening, commissural fusion, and annular deformation using obturators. If the annulus is dilated or deformed, the technique employs a remodeling approach using prosthetic rings, which are shaped and sized based on measurements of the anterior leaflet. This method selectively reduces the annulus at points of excessive dilation, particularly the commissures, while maintaining the valve’s shape to avoid stenosis [27].

The De Vega annuloplasty technique is a surgical procedure designed to treat functional TR by reducing and stabilizing the size of the tricuspid annulus. The procedure involves the placement of a double continuous suture along the dilated portions of the tricuspid annulus, mainly focusing on the anterior and posterior segments, which are prone to dilation. The sutures are carefully placed to avoid the septal portion of the annulus, thereby protecting the conduction system. This technique is favored due to its simplicity, effectiveness, and ability to be performed by less experienced surgeons, with low rates of reoperation for recurrent TR [28].

Both the Carpentier and De Vega annuloplasty techniques have been utilized as treatment options for TR in HT recipients. TA performed during the HT process has demonstrated efficacy in mitigating early postoperative TR. For example, a study comparing 25 patients who received donor heart TA with either the De Vega or Carpentier’s ring techniques to a cohort of 25 patients who did not undergo TA found that preemptive TA significantly reduced postoperative TR, as evidenced by lower TR scores on early echocardiograms [4]. However, TA in HT is also linked to a higher occurrence of conduction abnormalities, such as right bundle branch block (37% vs. 9% in non-TA patients) and complete heart block, resulting in more frequent pacemaker implantations. While TA effectively prevents severe TR, it does not offer significant long-term hemodynamic benefits [29]. The studies by Jeevanandam et al. address the debate over the timing and impact of surgical tricuspid valve repair in HT. Their 2004 study demonstrated that prophylactic donor TA improved renal function but did not significantly enhance survival rates [30]. In a follow-up 2006 study, the prophylactic approach was still beneficial in preventing severe TR, but it did not lead to substantial long-term improvements in overall survival or hemodynamics [31]. These findings suggest that while donor TA may have some short-term advantages, its long-term benefits in terms of survival and cardiac function remain uncertain. Prophylactic TA during HT can increase the risk of right heart failure, particularly when pulmonary arterial hypertension is present. This is because, without the natural “pressure relief” function of the TR, the RV may struggle to manage the increased pressure. Conversely, moderate to severe TR, coupled with pulmonary hypertension, can exacerbate RV dysfunction by leading to volume overload and eventual right heart failure [25, 32, 33].

### Surgical tricuspid valve replacement (sTVR)

sTVR has proven to be an effective approach for managing severe TR in HT recipients when medical therapy alone is inadequate, although based on evidence from case reports and small, single-center studies [34, 35]. In a study of 163 HT patients, nine individuals received TVR due to significant TR that developed an average of 5 years after the transplant. The treatment significantly reduced TR severity in all cases, leading to substantial clinical benefits, including an average 24% reduction in serum creatinine and a 47% decrease in total bilirubin. Patients also reported improved symptoms and reduced diuretic use [35]. These findings highlight TVR as a promising treatment for severe TR in post-transplant patients, although careful patient selection is crucial to minimize risks and optimize outcomes. In the study by Raghavan et al., the types of valves used for sTVR are not mentioned [35]. In contrast, the case report by Votapka et al. specifies the use of a Hancock porcine valve for sTVR [34]. Compared to biological valves, mechanical valves for TVR in HT recipients have several drawbacks, including the need for anticoagulation and the inability to perform EMBs.

### Interventional approaches

#### Transcatheter tricuspid valve repair (tTVr)

The complexity of the tricuspid valve anatomy, including the large annulus, dense chordae, and the challenging geometry of the valve in HT recipients, poses significant technical difficulties in the development and application of transcatheter therapies for TR. So far, the interventional treatment of TR in HT recipients is a field, with most available evidence derived from case reports and small case series. One of the earliest transcatheter techniques employed for managing TR was edge-to-edge repair using the MitraClip device. This approach was employed successfully until the introduction of dedicated transcatheter techniques specifically designed for TR.

Successful TR repair with the MitraClip was initially described in a 20-year post-HT patient, resulting in significant symptomatic improvement and TR reduction [36]. A subsequent report documented successful percutaneous treatment of severe TR in two elderly HT patients. Both patients had developed severe TR due to multiple EMBs that caused leaflet flail, and they were at high risk for sTVR. The tTVr procedure led to a significant reduction in TR, with normalization of right ventricular size and function. Both patients had excellent clinical outcomes, remaining stable and independent during a 2-year follow-up period [37].

In a case series of seven HT recipients (median age 53, predominantly female), transcatheter tricuspid valve edge-to-edge repair (T-TEER) was performed using the TriClip device. The procedure, which involved implanting 2–3 clips in a tricuspid position, was technically successful in six of the seven patients. In one patient, however, due to a single leaflet device attachment, a second clip was used to stabilize the detached leaflet. The intervention resulted in significant TR reduction and right ventricular remodeling at follow-up. Most patients were discharged within 2–4 days without in-hospital adverse events. During a median follow-up of 13 months, one patient died from non-cardiac causes (perforated diverticulitis with burst abdomen, compounded by a suspected acute rejection and right heart failure, despite the near-optimal reduction of TR), while the remaining six experienced improved NYHA functional class, suggesting that T-TEER is a promising, minimally invasive alternative for managing TR in this high-risk population [38]. A TriClip device was also used for percutaneous tricuspid repair in a 67-year-old HT patient with severe TR, resulting in a successful reduction of the regurgitation [39]. Another device is the PASCAL Ace system, which has been used successfully in two HT patients in an initial case series [40].

### Transcatheter tricuspid valve replacement (tTVR)

Currently, there are no reports on the use of tTVR systems, such as EVOQUE, in HT recipients. However, in the TRISCEND trial, elderly patients with multiple comorbidities and at least moderate TR demonstrated sustained TR reduction and significant improvements in cardiac output, along with high survival rates and reduced hospitalizations after tTVR [41]. HT patient recipients are often high-risk surgical candidates due to factors such as immunosuppressive therapy, prior surgeries, and various comorbidities, which make them less suitable for traditional surgical repair or replacement. While transcatheter interventions show promising early results, they remain relatively novel in HT recipients. Consequently, larger randomized studies are needed to further evaluate their long-term efficacy and safety in this special patient population.

## Conclusions

TR remains a complex challenge in the post-HT landscape, characterized by its multifactorial etiology and variable impact on patient outcomes. Despite the high prevalence of TR and its associated complications, the evidence supporting management strategies is limited and largely derived from case series. Current treatment approaches, including surgical techniques and emerging interventional methods such as edge-to-edge repair or transcatheter valve replacement, offer potential benefits for alleviating TR. While these approaches have shown some success in reducing TR severity and improving symptoms, their long-term efficacy and impact on patient survival remain uncertain. Addressing these gaps is essential for refining treatment strategies and ultimately improving patient outcomes in this challenging area.

## Limitations

Several limitations should be acknowledged in the current review. Firstly, the variability in the reported prevalence and severity of TR among studies reflects a lack of standardized diagnostic criteria and grading systems specific to HT recipients. Additionally, the evidence primarily relies on data from case reports, small case series, and single-center studies, which may not fully represent the broader transplant population and may have inherent biases. Long-term outcomes of the reported interventions require further investigation through larger, multicenter trials to establish their effectiveness and safety.

## Data Availability

No datasets were generated or analysed during the current study.
